# Changes in health and welfare after workers’ compensation benefits cease.

**DOI:** 10.23889/ijpds.v7i3.1974

**Published:** 2022-08-25

**Authors:** Daniel Griffiths, Michael Di Donato, Tyler Lane, Shannon Gray

**Affiliations:** 1 Monash University; 2 University of Toronto

## Objectives

To investigate welfare and health service use among workers with long-duration workers’ compensation claims after workers’ compensation stopped. To identify changes in health and welfare of workers whose compensation benefits ended due to a 260-week limit under s39(1) of the Workers’ Compensation Act New South Wales 2012 legislative amendments, Australia.

## Approach

Workers’ compensation claims from the New South Wales State Insurance Regulatory Authority were linked to records for social security payments, hospital and emergency department admissions, and health professional services. A cohort of 15,258 workers with long-duration workers’ compensation claims (>2 years) were classified based on whether compensation ended due to a 260-week limit (s39 group), or in circumstances where compensation benefits stopped independently of a 260-week limit (injured control group), and are contextualised with a community comparator (N=10,703). Changes in welfare and health service use were examined 12 months preceding, and 12 months following, a final workers’ compensation benefit payment.

## Results

After workers’ compensation benefits ceased under a 260-week limit there was a 53% increase in the uptake of social security benefits such as unemployment or disability payments by the s39 group, and levels of hospitalisation remained elevated compared to a community comparator. In contrast, workers whose compensation ended for other reasons, such as returning to work, saw a 28% increase in receipt of social security payments, and coincided with decreased hospitalisation incidence after exiting the workers’ compensation scheme. Overall, receipt of welfare and use of hospital health services after workers’ compensation ended was more common for people aged 65 or older, non-homeowners, single parents, and people living outside major cities. Social security payments were underrepresented for people with compensable psychological injuries, whilst hospitalisation was overrepresented.

## Conclusion

The introduction of a 260-week limit on workers’ compensation benefits resulted in a cohort of workers transitioning to the social security system, and did not coincide with a characteristic reduction in hospital service use. Policy changes must recognise interconnected consequences of changes to welfare and health. Transitional supports are encouraged.

